# Spreading Depression Sends Microglia on Lévy Flights

**DOI:** 10.1371/journal.pone.0019294

**Published:** 2011-04-26

**Authors:** Yelena Y. Grinberg, John G. Milton, Richard P. Kraig

**Affiliations:** 1 Department of Neurology and Committee on Neurobiology, The University of Chicago Medical Center, Chicago, Illinois, United States of America; 2 The Claremont Colleges, Claremont, California, United States of America; Cajal Institute, Consejo Superior de Investigaciones Científicas, Spain

## Abstract

Spreading depression (SD) is thought to cause migraine aura, and perhaps migraine, and includes a transient loss of synaptic activity preceded and followed by increased neuronal excitability. Activated microglia influence neuronal activity and play an important role in homeostatic synaptic scaling via release of cytokines. Furthermore, enhanced neuronal function activates microglia to not only secrete cytokines but also to increase the motility of their branches, with somata remaining stationary. While SD also increases the release of cytokines from microglia, the effects on microglial movement from its synaptic activity fluctuations are unknown. Accordingly, we used time-lapse imaging of rat hippocampal slice cultures to probe for microglial movement associated with SD. We observed that in uninjured brain whole microglial cells moved. The movements were well described by the type of Lévy flight known to be associated with an optimal search pattern. Hours after SD, when synaptic activity rose, microglial cell movement was significantly increased. To test how synaptic activity influenced microglial movement, we enhanced neuronal activity with chemical long-term potentiation or LPS and abolished it with TTX. We found that microglial movement was significantly decreased by enhanced neuronal activity and significantly increased by activity blockade. Finally, application of glutamate and ATP to mimic restoration of synaptic activity in the presence of TTX stopped microglial movement that was otherwise seen with TTX. Thus, synaptic activity retains microglial cells in place and an absence of synaptic activity sends them off to influence wider expanses of brain. Perhaps increased microglial movements after SD are a long-lasting, and thus maladaptive, response in which these cells increase neuronal activity via contact or paracrine signaling, which results in increased susceptibility of larger brain areas to SD. If true, then targeting mechanisms that retard activity-dependent microglial Lévy flights may be a novel means to reduce susceptibility to migraine.

## Introduction

Spreading depression (SD) is a paroxysmal perturbation of brain that is thought to cause migraine aura, and perhaps migraine [1]. It is classically defined as a transient loss in spontaneous and evoked electrical activity, associated with a large DC potential change in the interstitial space, which both propagate at a uniquely slow speed of about 3 mm/min [2], [3]. SD is triggered in susceptible gray matter areas of brain where a sufficient volume is synchronously depolarized [4]. This triggering effect results from increased excitation, reduced inhibition, or a combination of these two effects, which results in a flurry of spontaneous discharges that immediately precede the loss in activity of SD [5]–[7]. Furthermore, recent evidence showed that spontaneous and evoked activity is increased long after episodes of SD (Kraig lab unpublished observations).

Evidence indicates that microglia are activated by increased synaptic activity [8], [9] and that their signaling can also influence synaptic activity [10]–[13]. Stellwagen and coworkers show that TNF-α enhances neuronal excitation by increasing AMPA receptor cell surface expression and reducing GABA receptor membrane levels [12], [13]. Furthermore, Turrigiano and colleagues show that this capacity of microglia is involved in homeostatic synaptic scaling, an adaptive response of brain directed toward tuning neural circuit activity to a functionally optimal state [14]. Thus by extension, microglia are likely to be involved in SD. Indeed, SD activates microglia [15], [16]. However, whether activated microglia can in return affect SD susceptibility has not been explored.

Microglial motion reflects their activation state. Within the context of disease, microglia travel directionally toward sites of irreversible injury [17]. In contrast, within healthy brain tissue, microglial somata remain in place, but during increased synaptic activity their processes extend and retract at an increased rate [18]. Since SD is preceded by a flurry of increased synaptic activity [4], [6], [7] followed by a brief period of electrical silence during SD, [and then long afterwards, a persistent increase in synaptic activity (Kraig lab, unpublished observations)], we asked how SD might alter microglial motion.

The rationale for this line of questioning stems from the realization that immune cells are activated (and influence other cells) by contact mediated effects as well as by paracrine signaling. Accordingly, we probed for microglial cell motion associated with SD using vital imaging of microglia in mature rat hippocampal slice cultures. Our results show that a fraction of microglia in control slice cultures moved in a stereotypic fashion consistent with Lévy flights. Furthermore, hours after SD, the number of microglia moving long distances was significantly increased. We next asked whether this effect could be mimicked by alterations in synaptic activity. Synaptic activity increased by activation of microglia [with lipopolyssacharide (LPS)] as well as neuronal activity increased by chemical long-term potentiation (cLTP) significantly decreased the number of microglia moving long distances. In contrast, blockade of synaptic activity via exposure to tetrodotoxin (TTX) significantly increased the number of microglia moving long distances and this increase could be abrogated by co-incubation with glutamate and adenosine triphosphate (ATP), two paracrine mediators released with synaptic activity, for which microglia have receptors.

Recently the study of cell movements from the perspective of a random walk has attracted great interest [19]–[25]. These studies have focused on the movements of cells in culture or over surfaces. Here we provide the first evidence to show that microglia travel via Lévy flights. Furthermore, this is the first evidence that any cells within a living tissue move via Lévy flights. Moreover we show that these movements correspond to the type of Lévy flight that has been associated with an optimal random search pattern [26]–[28]. We suggest microglial migration after SD may be a means by which these cells influence a wider expanse of brain either by contact or by paracrine signaling, perhaps to increase regional susceptibility to SD, and by extension, migraine.

## Methods

### Tissue preparation and maintenance

We chose rat hippocampal slice cultures to study microglial movement because electrophysiological function [7], [29] and astrocyte reactive state [30] are like that seen *in vivo*. Furthermore, microglia are quiescent in hippocampal slice cultures [16], in which precise control over environmental conditions can be maintained, thus being ideal for the study of low-level immune signaling [29], [31], [32].

This study was carried out in accordance with the recommendations in the Guide for the Care and use of Laboratory Animals of the National Institutes of Health. The project was reviewed and approved by the University of Chicago Institutional Animal Care and Use Committee (active protocol #51511).

Rat hippocampal slice cultures were prepared as previously described [30]. In brief, hippocampi collected from P8–10 rat pups were sliced to 350 µm. These slices were then kept in 6-well Falcon trays (#08-772-1B; Thermo Fisher) on Millicell-CM tissue culture inserts (#PICM0-3050; Thermo Fisher) at 36°C with 5% CO_2_-balance air. Cultures were kept in horse serum (HS) media, which was changed biweekly, throughout all procedures. HS media (per 100 ml) consisted of: 50 ml Basal Medium Eagle (#21010; Invitrogen), 25 ml Earle's Balanced Salt Solution (#E2888; Sigma), 23 ml horse serum (#26050-088; Invitrogen), 0.5 ml Glutamax™ (at 200 mM; #35050; Invitrogen), 0.1 ml Gentamicin (at 10 mg/ml; #15710-064; Invitrogen), 0.4 ml Fungizone (at 250 µg/ml; #15295; Sigma), 1.45 ml D-Glucose (42 mM; #G8769; Sigma).

Slice cultures were screened for cell death at 18 days *in vitro* (DIV) with 5 µM Sytox Green (504/523 nm fluorescence excitation/emission when bound to DNA; #S7020; Invitrogen) [16]. Cultures with pyramidal neuron layer injury were not used. Cultures were used between 18 and 35 DIV, a period associated with mature synaptic function and quiescent microglia consistent with those seen normally *in vivo* [7], [16], [33].

All chemical modulators were added immediately prior to imaging. Drug doses were 10 µM tetrodotoxin (TTX; #T5651; Sigma), 29 µg/ml LPS (#L4774; Sigma), 5 mM ATP (#A1852; Sigma), 5 mM glutamate (#G1626; Sigma). We used a well-accepted method for inducing cLTP in slice cultures that uniformly activates a wide area of CA1 [34]–[36]. This method elevates cAMP levels and increases synaptic activity using rolipram (100 nM; #R6520; Sigma) and forskolin (50 µM; #F3917; Sigma) applied in a Mg^2+^ - free Ringer's solution (36°C) for 10 min followed by dipping inserts in fresh media (36°C) three times before filming. Our results (not shown) indicate that this cLTP procedure does not result in pyramidal neuron excitotoxic injury when slices were screened 24–72 hours after cLTP.

**Figure 1:**
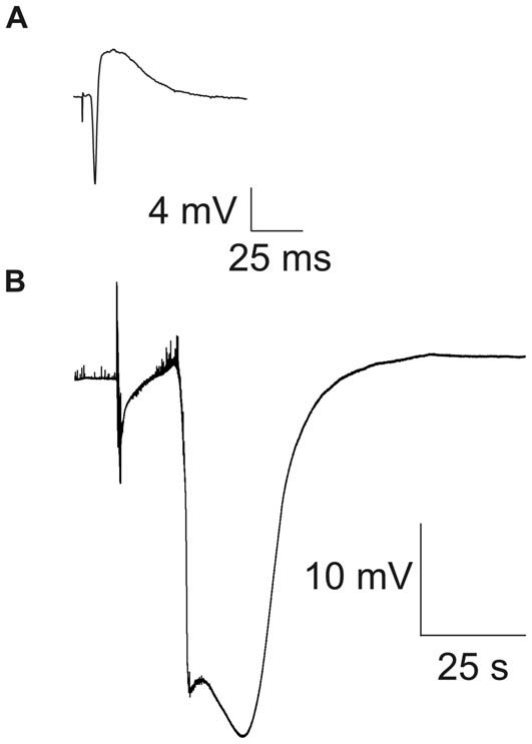
Spreading depression occurs with a transient loss of synaptic activity. (**A**) Slice electrophysiological responsiveness was first verified by eliciting standard CA3 field potentials evoked by dentate gyrus bipolar electrical stimulation. Record shows typical CA3 evoked field potential with downward deflection from CA3 action potential and upward deflection, the “inverted” excitatory post-synaptic potential, recorded at the pyramidal neuron cell body area. (**B**) Spreading depression (SD) was elicited every ∼9 min for 1 hour by dentate gyrus bipolar electrical stimulation and confirmed via the stereotypic large negative DC potential change in the extracellular space. Small deflections in baseline record reflect spontaneous neuronal activity, which after the stimulus pulse for SD (first large vertical deflections in record) was followed by increased spontaneous activity (evident as increased baseline deflections). The latter led to the massive DC change of SD and thinning of the DC line (consistent with electrical silence).

SD was elicited every ∼9 min for 1 hour by dentate gyrus bipolar electrical stimuli and confirmed via extracellular field potential recordings (**Fig. 1**) in the CA3 pyramidal neuron layer as previously described [16] with minor modifications. SD can not be triggered reliably in HS-based media [16]. Accordingly for SD, slices were transferred to a serum-free media (per 100 ml) that consisted of: 97 ml Neurobasal (#21103; Invitrogen), 2 ml Gem-21 (#400-160; Gemini Bio-Products), 0.5 ml Glutamax™ (at 200 mM), 0.1 ml Gentamicin (at 10 mg/ml), 0.4 ml Fungizone (at 250 µg/ml), 1.45 ml D-glucose (42 mM) [37], [38] supplemented with 41 mM NaCl (#S6546; Sigma), 1.6–2.4 mM CaCl_2_ (#GA10984; Sigma), and 0.8 mM MgCl_2_ (#057K8620; Sigma) and held in a static perfusion format. Chamber temperature was maintained at 36°C with 5% CO_2_-balance air aeration to maintain media at pH 7.3.

We measured changes in excitability after SD by measuring field potential responses to single paired pulse inhibition (PPI) using standard procedures but were sure to keep stimulation parameters constant [39]. Briefly, CA3 evoked field potentials were evoked by bipolar dentate gyrus stimulation (100 µs, 0.2 Hz) and optimized by first beginning with a current that was sufficient to trigger a response and then moving the recording microelectrode perpendicular to the mid CA3 pyramidal neuron layer until the response was maximal, followed by changing the stimulus current (i.e., typically 10–20 µA) until the evoked CA3 response was half-maximal. PPI consisted of eliciting equivalent paired pulses 20 ms apart at half-maximal stimulus intensity [39].

### Staining procedure

AlexaFluor594-tagged Isolectin GS-IB_4_ (590/617 nm fluorescence excitation/emission; #I21413; Invitrogen) was added 3–24 hours before the start of filming. 20 µl of 1 mg/ml stock was added to 1.1 ml of media for each slice culture insert. Prior to and immediately after imaging, slices were screened for cell death using Sytox Green. After exposure to Sytox Green, inserts were rinsed 3 times in Neurobasal before imaging.

### Imaging procedure

**Figure 2:**
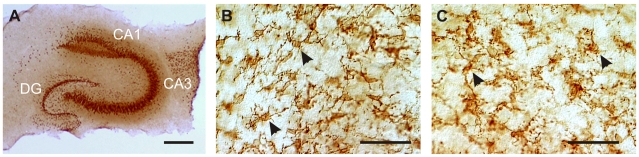
Hippocampal slice microglial morphology was not altered by exposure to isolectin GS-IB_4_. (**A**) For cytoarchitectural reference, we show hippocampal principal neuron architecture using anti-NeuN immunostaining to illustrate the CA3 pyramidal neuron area. Microglia were immunolabeled specifically with CD11b to visualize their morphology under control conditions (**B**) and after 3 hour exposure to isolectin GS-IB_4_ (**C**). In both microglial exemplary images, the cells (arrows) show a ramified morphology without evidence of activation (i.e., short and thick branches or change to ameboid cellular shape). Cal bars, 500 µm (**A**) 50 µm (**B** and **C**).

**Figure 3:**
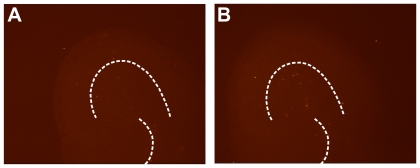
Imaging procedures did not result in cell death. Slice cultures were screened for vitality via the fluorescent dead-cell marker Sytox Green immediately before and after imaging. The dotted large arc outlines the pyramidal neuron layer and the smaller arc outlines the dentate gyrus. (**A**) An exemplary image acquired before filming showed no evidence of cell death. (**B**) Similarly, no evidence of cell death was seen after filming. These pre- and post-imaging Sytox pictures correspond to the control movie used for **Figure 4** and **Supplemental Video S1**.

MetaMorph software (version 7.5.4.0; Molecular Devices) was used to acquire one in-focus image every minute for 6 hours. An in-focus image centered on the CA3 pyramidal neuron layer was acquired each time by first acquiring a stack of 8 images, 1 µm apart, with the best-focus image kept as the frame for that minute-frame and its Z-axis location set as the new Z “home.” Using this procedure, tissue was exposed to 480–600 ms of UV light per minute, with a 2.0 neutral density filter dampening the intensity of the light from a 100 W Hg vapor lamp. This time-lapse imaging protocol was used to minimize light exposure, and thus potential phototoxicity, and to allow a sufficient window of observation to detect multiple microglial movements. Phase images were obtained at 5x, 10x, and 20× prior to time-lapse imaging to register microglial imaging referable to the CA3 pyramidal neuron layer. Imaging procedures did not trigger microglial activation (**Fig. 2**) or cell death (**Fig. 3**).

### Imaging set-up

Immediately prior to imaging, culture inserts were placed into 35 mm dishes fitted with 1 mm glass rods on their bottom, stabilizing the insert membrane from vertical movement. Three 2 mm pieces of tubing (#95809; Pharmed) were placed equidistantly around the insert to prevent horizontal movement. Absorbent cotton strips (0.01×3 cm) were soaked in 1 ml culture media and fitted to the walls inside the insert away from the hippocampal slices, to add humidity. The culture dishes and insert, once assembled with media and cotton, were covered with polyvinyl chloride film (Fisher Scientific) in order to allow for CO_2_ diffusion and prevent fluid loss. The slice culture assemblies were then placed in a microincubator (#PDMI-2; Medical Systems) aerated with 5% CO_2_-balance air, at 36°C and pH maintained at 7.3, verified using a micro-combination pH electrode (#M-413; Microelectrodes). Images were acquired on a Leica DMIRE2 microscope using QuantEM:512SC camera (Photometrics) and a LAMBDA SC Smart Shutter™ (Sutter Instrument).

### Analysis and quantitation

MetaMorph software's Track Objects feature was used to quantitate microglial movement. Moving cells in each movie were first identified by visual inspection. Cells that moved a distance ≥50 µm from their starting position (termed *origin* in MetaMorph) were tracked. Microglia are approximately 50 µm in diameter when including branches [40] thus movements ≥50 µm (see below) were defined as whole cell movement. Tracking parameters were object region (7x7 µm) and search region (10x10 µm). Distance from origin, x- and y-coordinates, as well as an image of the path traveled, were registered for each of the tracked cells.

We tracked microglial movement in rat hippocampal slice cultures for 6 hours without injury or microglial activation from dye exposure (**Figs. 2** **&** **3**). Visual inspection showed that while microglial processes moved vigorously, as reported in anesthetized mouse neocortex *in vivo* [18], [41], whole cells in unanesthetized hippocampal cultures, which are ideal for study of microglia [31], [32], were able to move and did so in a stereotypic pattern (**Supplementary Figure S1 and Supplementary Video S1**).

### Data Analysis

**Figure 4:**
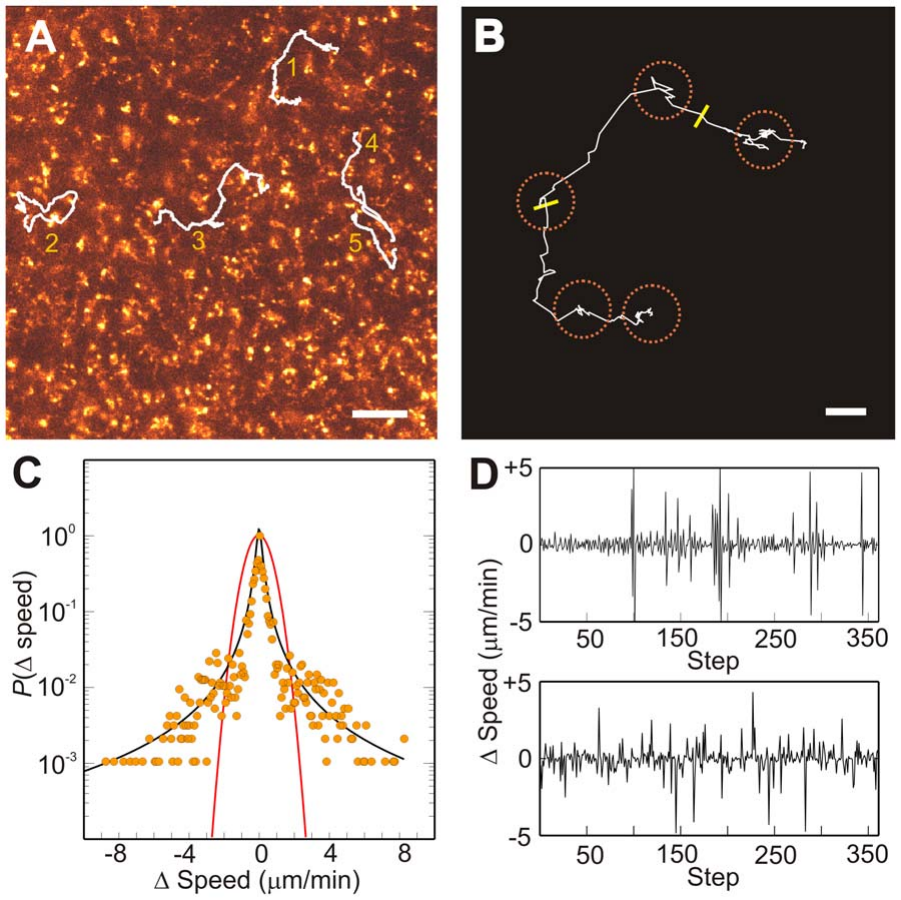
Microglia moved long distances in a Lévy flight pattern. (**A**) Control movie frame with white lines showing paths of microglia (1–5) that moved ≥50 µm. (**B**) Cell #1 (**A**) path enlarged to show movement beginning at upper right and ending at lower left, yellow bars mark 2 and 4 hours. Orange dashed lines encircle regions with multiple small steps compared to occasional long steps, suggestive of a Lévy flight pattern. (**C**) Control microglial cell Δ velocity (*n* = 18 cells; 6462 Δ's) showed a Lévy power law distribution with exponent α = 0.9 and scale factor γ = 0.3 (black line). The red line shows a Gaussian distribution (α = 2; variance = 0.5). (**D**) Realization of a Lévy flight constructed using the distribution (**C**) determined from the full population of moving microglia (top) qualitatively reproduced the same intermittent behavior of an exemplary long-distance moving microglial cell (bottom). Cal bars, 50 µm (**A**) and 10 µm (**B**).



The movements of all microglia cells within view that moved more than one cell diameter (50 µm) [39] in 6 hours were analyzed. The distance, 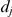, traveled by the j-th microglia in the (x,y)-plane during one time step is
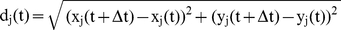
Thus the magnitude of the velocity, or speed, 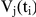 is
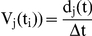
where 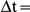 1 min. The change in speed, 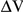, is
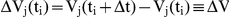
The quantity 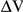
does not correspond to the acceleration since we do not consider the direction of the microglial movement, but is equivalent to a high pass filtering of the time series [42]. Our goal was to test the hypothesis that the movements of microglia are governed by a Lévy flight, i.e., a random walk for which the 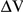 are chosen from the power law distribution [26]–[28]

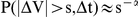
where α is the Lévy index. Thus a Lévy flight consists of a random walk characterized by many small 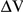
with occasional relatively large 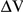 as we observe for microglia (**Figs. 4A & 4B**) and as predicted to occur for moving cells [21]. After many steps the probability distribution 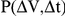 converges to the Lévy stable distribution

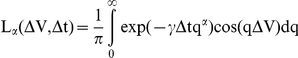
where γ is a scale factor. For a simple random walk the limiting probability distribution is the normal, or Gaussian, distribution which is, in turn, a Lévy distribution with 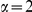 and variance 2γ.

We combined the individual 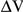 measured for all moving microglia and fit the distribution of 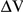 to a Lévy distribution using a program, stblfit.m, obtained from a MATLAB software package developed by M. Veillette (Boston University) which is freely downloadable from the Internet site: http://math.bu.edu/people/mveillet/html/alphastablepub.html. The description of a Lévy distribution provided by stblfit.m is in terms of four parameters: the Lévy, or characteristic, exponent (α); the skewness; the scale factor (γ); and the location. However, both the skewness and location factors were approximately 0 for distribution fits involving 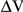. A Lévy random number generator, stblrnd.m, also supplied by the above MATLAB software package, was used to generate a series of Lévy variables 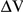 drawn from the Lévy distribution specified by α and γ. To compare a realization of this series (**Fig. 4D**, top) to the 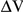 measured for a single microglial cell (**Fig. 4D**, bottom), it was necessary to truncate the probability distribution in order to account for the fact that the speed of microglial movements is finite. We did this by cutting off those values generated by stblrnd.m which were greater or smaller than a threshold estimated from the movements of the microglia.

### Statistical Procedures

**Figure 5:**
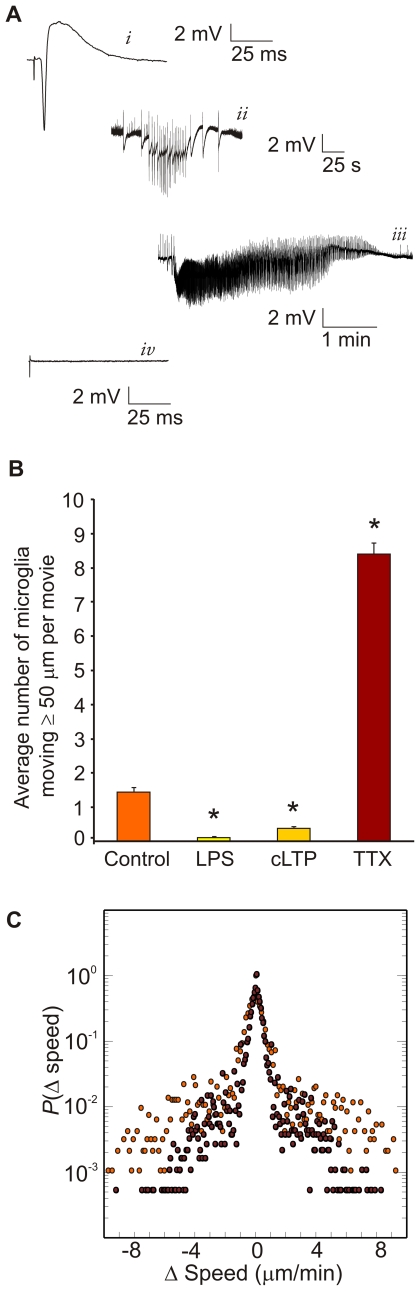
Microglial long-distance movement was inversely proportional to neuronal activity. (**A**) Control slice cultures showed typical spontaneous (not shown) and evoked (*i*) CA3 area field potentials. Slices exposed to lipopolysaccharide (LPS) or chemical long-term potentiation (cLTP) showed increased synaptic activity that included CA3 area bursting (*ii*) and electrographic seizures (*iii*). Slices exposed to tetrodotoxin (TTX) showed no spontaneous or evoked synaptic activity (*iv*). Time calibration bars are 25 msec (*iv*) and 25 sec (*v*). (**B**) Microglial movement was quantified by tracking the cells that moved ≥50 µm from their position of origin. Specific microglial cell counts were 1.44±0.43 for control (*n* = 16 movies; range: 1–5 cells/movie), 0.06±0.06 for LPS (*n* = 16 movies; range: 0–1 cells/movie), 0.38±0.15 for cLTP (*n* = 16 movies; range: 0–2 cells/movie), and 8.40±1.89 for TTX (*n* = 5 movies; 3–13 cells/movie). Values are mean ± SEM. Data was statistically tested (see text) using cell counts transformed by √(*n*+1) with significance (P<0.05; “*”) shown here for illustration. (**C**) Microglial movement after TTX exposure also showed a Lévy power law distribution (red dots; *n* = 42 cells; 15,523 Δ's). The Lévy distribution of control cells (orange dots) is included for comparison, to show that the distributions appear very similar.

Microglial cell movement data (**Figs. 5** **–** [Fig pone-0019294-g006] **7**) was compared for significance using mean ± SEM of cell counts transformed via √(*n*+1) because of low cell counts [43]. In addition, controls (*n* = 16 movies) were set to 1.00 with related group results scaled proportionally to facilitate inter-experiment interpretations. Multiple comparisons were tested using ANOVA plus post hoc Holm-Sidak and differences between groups tested via two-tailed Student's *t*-test.

**Figure 6 pone-0019294-g006:**
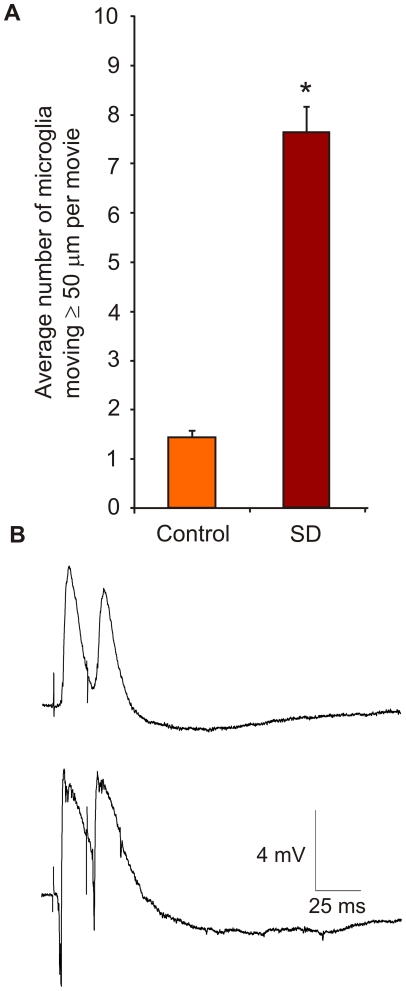
Microglial migration increases long after the electrical silence associated with spreading depression. (**A**) Microglial movement was recorded 7–13 hours after induction of spreading depression (SD) and microglial cell movement was quantified as with the other conditions, by tracking the cells that moved ≥50 µm from their position of origin. Specific microglial cell counts for SD movies (*n* = 5) were 0–16 cells per movie with a mean ± SEM of 7.60±2.86 cells/movie. Cell counts were 1.44±0.43 for control (*n* = 16 movies; range: 1–5 cells/movie). Values are mean ± SEM. Data was statistically tested (see text) using cell counts transformed by √(*n*+1) with significance (P<0.05; “*”) shown here for illustration. (**B**) Exemplary records of field potential responses to single paired pulse inhibition (PPI) show increased excitability following SD. Before SD (upper record) the second evoked response is decreased, consistent with fast GABAergic inhibition. The latter is not evident with PPI responses evoked 9 hours after SD (bottom record).

**Figure 7:**
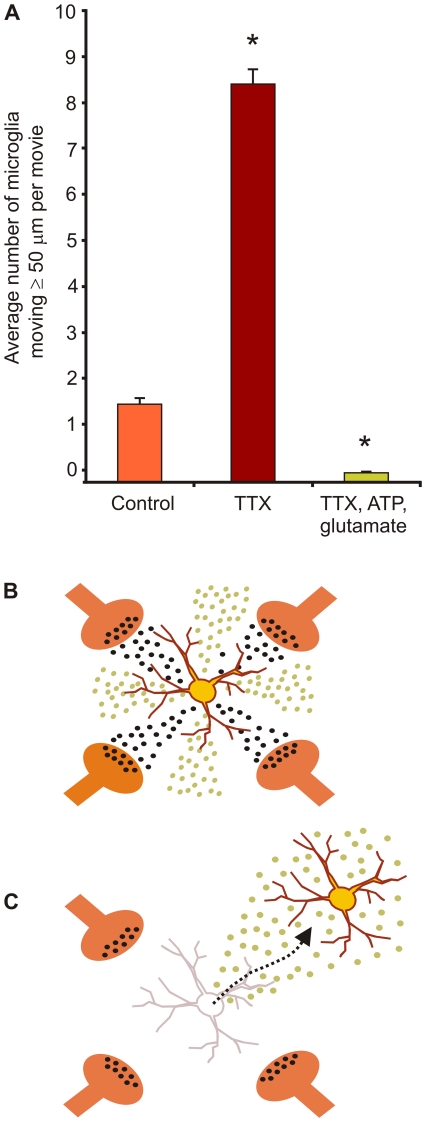
Schematic of potential interactive paracrine basis of microglial cell movement. (**A**) We added ATP and glutamate to hippocampal cultures in TTX and observed that this paradigm abrogated the increased microglial movement normally seen with TTX alone. Data was statistically tested (see text) using cell counts transformed by √(*n*+1) with significance (P<0.05; “*”) shown here for illustration. (**B**) Cartoon schematic shows how paracrine signals (e.g. glutamate or ATP) released from neurons with synaptic activity (black) may signal microglial cells to remain stationary (top) where they can respond with release of neuronal activity modulators (e.g. TNF-α [green]). In contrast, reduced neuronal activity may allow for the “release” of microglial cells to wander (bottom), thus allowing them to affect larger expanses of brain.

## Results

### GS-Isolectin-B4 does not activate microglia

TNF-α protein was quantified in slices exposed to isolectin compared to control as a measure of microglial activation. Slice cultures were exposed to isolectin for 3–24 hours and harvested for TNF-α measurement via microsphere-based flow cytometric immunoassay [29]. Results showed no significant difference (*P* = 0.71; two-tailed Student's *t*-test) between isolectin-treated slice cultures (13±1 pg/mg brain; *n* = 4) and controls (14±2 pg/mg brain; *n* = 4). We also examined the morphology of microglia after isolectin exposure versus control using cell-specific immunostaining for CD11b (*n* = 3/group). We found no evidence of ameboid transformation in microglial shape consistent with their transformation to an activated state. Also, branch morphology was comparable between the two groups, further indicating their comparable normal quiescent states (**Fig. 2**).

### Characterizing Microglial Movement

Microglia movements were characterized by long intervals during which the cells moved very slowly interspersed with brief intervals during which they moved more quickly (**Fig. 4A & 4B**). This observation is suggestive of a Lévy flight. **Figure 4C** shows that the distributions of the 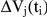 obtained by pooling data for *n* = 18 microglia was symmetrically distributed about 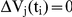. A striking feature is the presence of 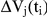 larger than would be anticipated if it was assumed that the 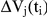 were distributed as a normal distribution (red line). The increased dispersion in 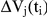 is readily accounted for by a Lévy distribution with 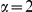 and 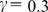. These observations suggest that the movements of the microglia do not resemble those of a simple random walk, but are those of a Lévy flight. **Figure 4D** shows that the intermittent pattern of the changes in ΔV for a realization of a Lévy flight from this measured distribution is qualitatively similar to that observed for a microglial cell. Thus, these observations demonstrate that microglial movements are much better described by Lévy flight, a distribution with “fat tails”, than by a simple random walk. Since α ∼1, microglial movements resemble those of an optimal random search pattern [28]. Lévy flight-type cellular movements can arise from a “stick-slip” locomotory pattern where cells move by protruding their front and retracting their rear [21]–[23], [44]. To our knowledge, this is the first evidence of cells moving via Lévy flights within tissue.

We next explored the relationship between microglial movement and synaptic activity and observed that microglial cell movement is inversely proportional to neuronal firing (**Fig. 5** **)**. We used LPS (**Supplementary Figure S2 and Supplementary Video S2**), which activates microglia and induces seizure-like bursting, as well as cLTP (**Supplementary Figure S3 and Supplementary Video S3**) to stimulate increased synaptic activity. Both LPS and cLTP increased synaptic activity including bursting and electrographic seizures (**Fig. 5A**). LPS and cLTP significantly reduced microglial cell movement (*P*<0.001 and 0.01; 1-β = 0.86; *n* = 16 movies/group; **Fig. 5B**). In contrast, when we abolished synaptic activity with TTX (**Supplementary Figure S4 and Supplementary Video S4**), which was electrophysiologically confirmed to induce electrical silence (**Fig. 5A**), we observed that microglial cell movement was significantly increased (*P*<0.001; 1-β = 0.99; *n* = 5) (**Fig. 5B**). Furthermore, detailed analysis of microglial movements from TTX exposure suggest these movement patterns also resemble Lévy flights (i.e., α = 1.1; γ = 0.2; *n* = 42 cells) (**Fig. 5C**).

Similarly, hours after transient reduction in neural activity from SD, microglial long-distance movement was significantly increased (**Fig. 6A** **; Supplementary Figure S5 and Supplementary Video S5**), a time when evoked inhibitory synaptic signaling was decreased (**Fig. 6B**). Finally, we mimicked synaptic signaling in the absence of electrical activity by adding ATP and glutamate to hippocampal slice cultures incubated in TTX (**Supplementary Figure S6 and Supplementary Video S6**). We found that the increased microglial movement otherwise seen in the presence of TTX was abrogated by co-administration of glutamate and ATP, significantly reducing movement (*P*<0.006; 1-β = 0.83; *n* = 5 movies; **Figs. 5** **&** **7a**).

## Discussion

We imaged microglia in healthy brain and found that hours after SD the cells moved long distances and in a stereotypical movement pattern consistent with Lévy flight. Through chemical modulation of neuronal activity we also showed that microglial movement was otherwise inversely proportional to synaptic activity. Application of exemplary neurotransmitters glutamate and ATP revealed that microglial movement could then be blocked in otherwise electrically silent tissue.

Microglia have recently been described to have highly motile branches that sample adjacent brain more frequently as synaptic activity increases and vice versa [18], [41]. These findings come from *in vivo* two-photon studies of transgenic mice with green fluorescent protein expressing microglia, which show that microglial cell bodies remain stationary while their branches are highly dynamic.

Microglial whole-cell migration has previously only been described in models of irreversible injury in leech nervous system where whole microglial cells move rapidly toward the site of injury [17]. There is currently no data, to our knowledge, showing long-distance microglial whole cell movement in uninjured brain.

The absence of this observation is perhaps due to insufficient sampling. In our control experiments, moving microglia comprised on average 3% of the total population. Our protocol allows for a large spatial and temporal window of observation, with approximately 64 microglial cells being observed with each time-lapse movie (with n>50 movies) for a continuous six hours each. Thus our protocol is longer and includes many more microglial cells than other studies involving microglial imaging, perhaps explaining why we were able to find migrating microglia to be a recurring event instead of an anomaly.

Ours is the first evidence of cells within any tissue moving via Lévy flights. It has been suggested that Lévy flight-type cell movement patterns can arise from a random stick-slip process that generates a more or less irregular mode of movement [21], [22]. What is surprising is that the movements of microglia are described by the special type of Lévy flight (e.g., α∼1) that is associated with an optimal random search [26], [27], [28]. Lévy flights have previously been observed to be performed by many species of animals, from single-celled eukaryotes to large mammals foraging for food. Microglial movement via this optimal search strategy may be related to their function as immune cells of the brain.

Immune cell translocation includes two purposes. The first is to form cell-cell contacts. Neurons present a large pool of membrane ligands such as CD200 and CD47, for which microglia have surface receptors [45]. Membrane-bound receptor-ligand pairs on neurons and microglia necessitates that microglia will come into direct contact with neurons. The second is to signal via paracrine mediators to facilitate further immune responses or modulate the function of surrounding cells. Resident macrophages of other tissues, such as the Kupffer cells of liver and osteoclasts of bone, migrate to influence the function of their resident tissue [46], [47].

Microglia can exert their effects on brain tissue through non-immune paracrine signaling. They release neurotrophins such as brain-derived neurotrophic factor (BDNF) [48]. Microglia also have receptors for many neurotransmitters, including glutamate and γ-aminobutyric acid (GABA) [9], [45]. Microglia thus are responsive to paracrine signaling specific to their neural environment, which expands their role within brain tissue beyond classical immune cell signaling. Furthermore, microglial cytokines, traditionally recognized as immune signals, also have tremendous potential to affect synaptic activity. For example, the cytokine TNF-α increases α-amino-3-hydroxyl-5-methyl-4-isoxazole-propionate (AMPA) and decreases GABA(A) receptors on neuronal membranes, thus increasing neuronal excitability [10], [12], [49]. Additionally, TNF-α is necessary for synaptic scaling [13], [14]. Microglial cells are thus able to receive and produce neuron-specific signals, and neurons have multiple means of responding to as well as expressing immune signals.

The involvement of microglial movement in SD's effects on brain tissue's increased excitability is currently unclear; however, their wandering through brain tissue could affect nearby cells in two ways (**Fig. 7B**). The first is through a change in paracrine signaling of cytokines, which could decrease in the regions where the microglial cell is no longer present and increase in the microglial cell's new location. Microglial movement to a new location may be important to understanding how movement of these cells could increase susceptibility of brain to subsequent SD. While TNF-α increases neuronal excitability via increased AMPA receptor (and decreased GABA receptor) function within minutes [12], BNDF, released from microglia, has a more delayed but similar impact on neuronal excitability. BNDF can reduce inhibition by reversing the neuronal chloride gradient, and thus converting GABA from an inhibitory to an excitatory signal [49]. It takes hours for the K^+^-Cl^−^ cotransporter, KCC2, protein levels to significantly decrease following BDNF application [50]. Importantly, our data show that both of these potential, and exemplary, means for wandering microglia to enhance neuronal excitability after SD occur when the movement of these cells is no longer inversely correlated to synaptic activity. Instead, microglial movement after SD is enhanced when synaptic activity is elevated. This suggests that, while the stimulus from reduced synaptic activity of SD needed to send microglia wandering is brief, evidence of their wandering is prolonged. That is: microglial cell motility subsequently occurs, paradoxically, during the *increased* synaptic activity that may be a result of the reversal of the neuronal chloride gradient.

Perhaps the signaling associated with the dampening of electrical activity during SD results in a protracted effect on microglial movement, leading to a long-lasting, maladaptive impact on neuronal excitability evident later. Microglial whole-cell movement hours following SD could thus be a functional initiating step to the subsequent increased excitability of enlarged expanses of tissue they survey, an effect consistent with the protracted structural changes observed in these cells days after SD [15]. Taken together these results suggest that microglial movement may be part of the long-lasting response of brain tissue to SD and could thus be a target for future work examining the role of microglia in SD susceptibility and by extension migraine.

## Supporting Information

Figure S1 and Video S1 **Control movie used in** **Figure 1** **.** All movies are imaged at 400×400 µm, over the CA3 pyramidal layer of rat hippocampal slice cultures. All movies were made with an image acquired every minute for 6 hours, for a total of 361 frames. Supplementary Figure S1 shows time zero frame of microglia to the left and matching phase image to the right with dotted lines marking the CA3 pyramidal neuron layer.
(TIF, M2V)

Figure S2 and Video S2 **Effect of increased neuronal activity upon microglial movement: LPS.** Here, Lipopolysaccharide (LPS) was added to the media 1 hour before time-lapse movie acquisition. LPS added to media increased neuronal excitability as shown in **Figure 5**. Supplementary Figure S2 shows time zero frame of microglia to the left and matching phase image to the right with dotted lines marking the CA3 pyramidal neuron layer.
(TIF, M2V)

Figure S3 and Video S3 **Effect of increased neuronal activity on microglial movement: cLTP.** One hour before the start of the movie, the insert was placed in chemical long-term potentiation (cLTP) media. This cLTP protocol increased excitability as shown in **Fig. 5**. Supplementary Figure S3 shows time zero frame of microglia to the left and matching phase image to the right with dotted lines marking the CA3 pyramidal neuron layer.
(TIF, M2V)

Figure S4 and Video S4 **Effect of decreased neuronal activity upon microglial movement: TTX.** Here, TTX was added to the media one hour before the start of the movie and was maintained throughout imaging. Supplementary Figure S4 shows time zero frame of microglia to the left and matching phase image to the right with dotted lines marking the CA3 pyramidal neuron layer.
(TIF, M2V)

Figure S5 and Video S5 **Effect of decreased neuronal activity upon microglial movement: Spreading depression.** Spreading depression was induced 7 hours before the start of the movie. Supplementary Figure S5 shows time zero frame of microglia to the left and matching phase image to the right with dotted lines marking the CA3 pyramidal neuron layer.
(TIF, M2V)

Figure S6 and Video S6 **Addition of ATP and glutamate to TTX to mimic synaptic signaling.** TTX was applied in the same manner as in the TTX-only movies, here with the addition of ATP and glutamate. Supplementary Figure S6 shows time zero frame of microglia to the left and matching phase image to the right with dotted lines marking the CA3 pyramidal neuron layer.
(TIF, M2V)
