# Supplementary figures and images for: Spreading Depression Sends Microglia on Lévy Flights
Source: PLoS One. 2011 Apr 26;6(4):e19294. doi: 10.1371/journal.pone.0019294 (PMC3082564; doi:10.1371/journal.pone.0019294)

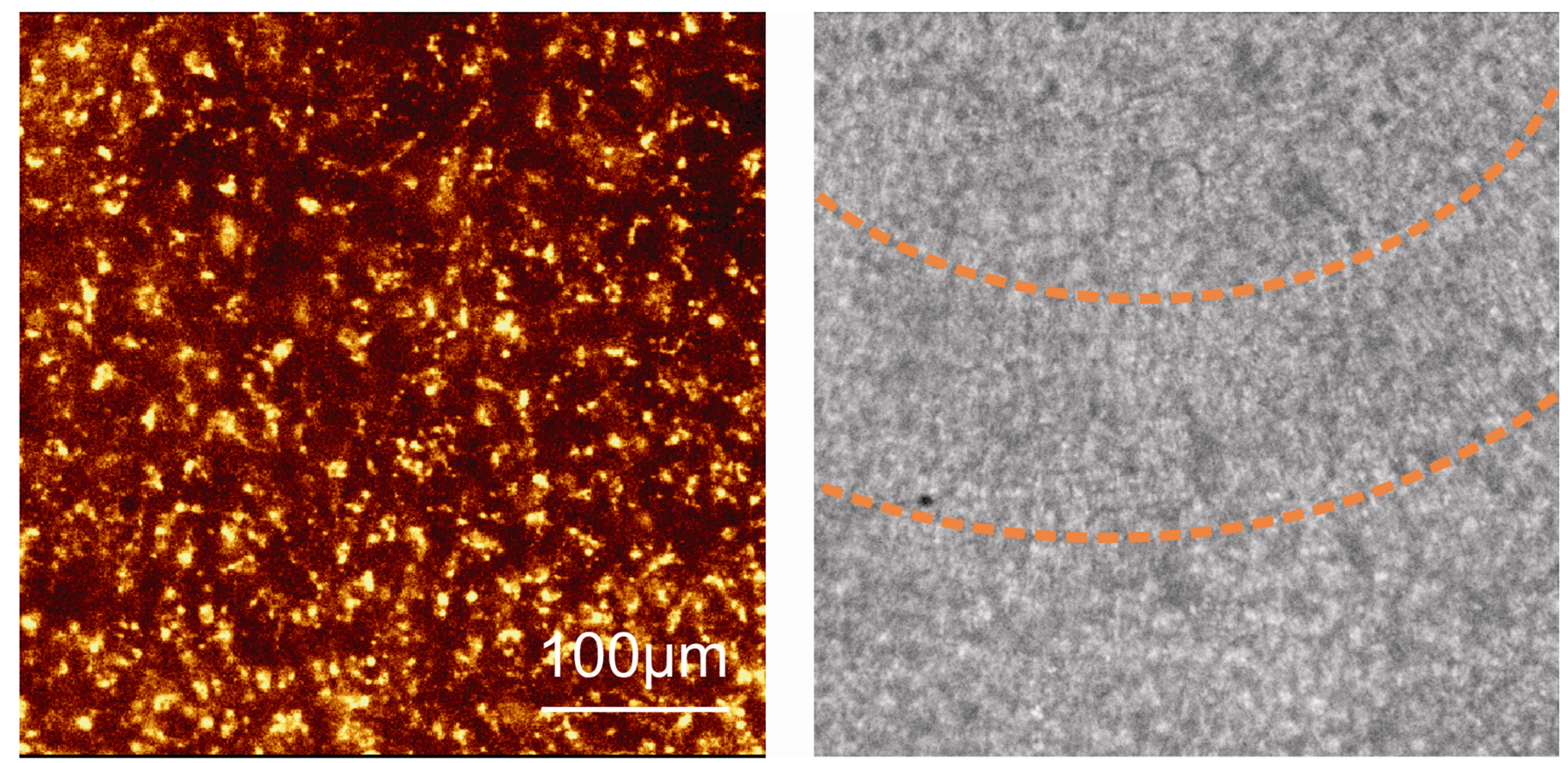

Supplement: Figure S1 and Video S1 — Control movie used in Figure 1 . All movies are imaged at 400×400 µm, over the CA3 pyramidal layer of rat hippocampal slice cultures. All movies were made with an image acquired every minute for 6 hours, for a total of 361 frames. Supplementary Figure S1 shows time zero frame of microglia to the left and matching phase image to the right with dotted lines marking the CA3 pyramidal neuron layer. (TIF, M2V) [file pone.0019294.s001.zip › Supplementary Figure 1.tif]

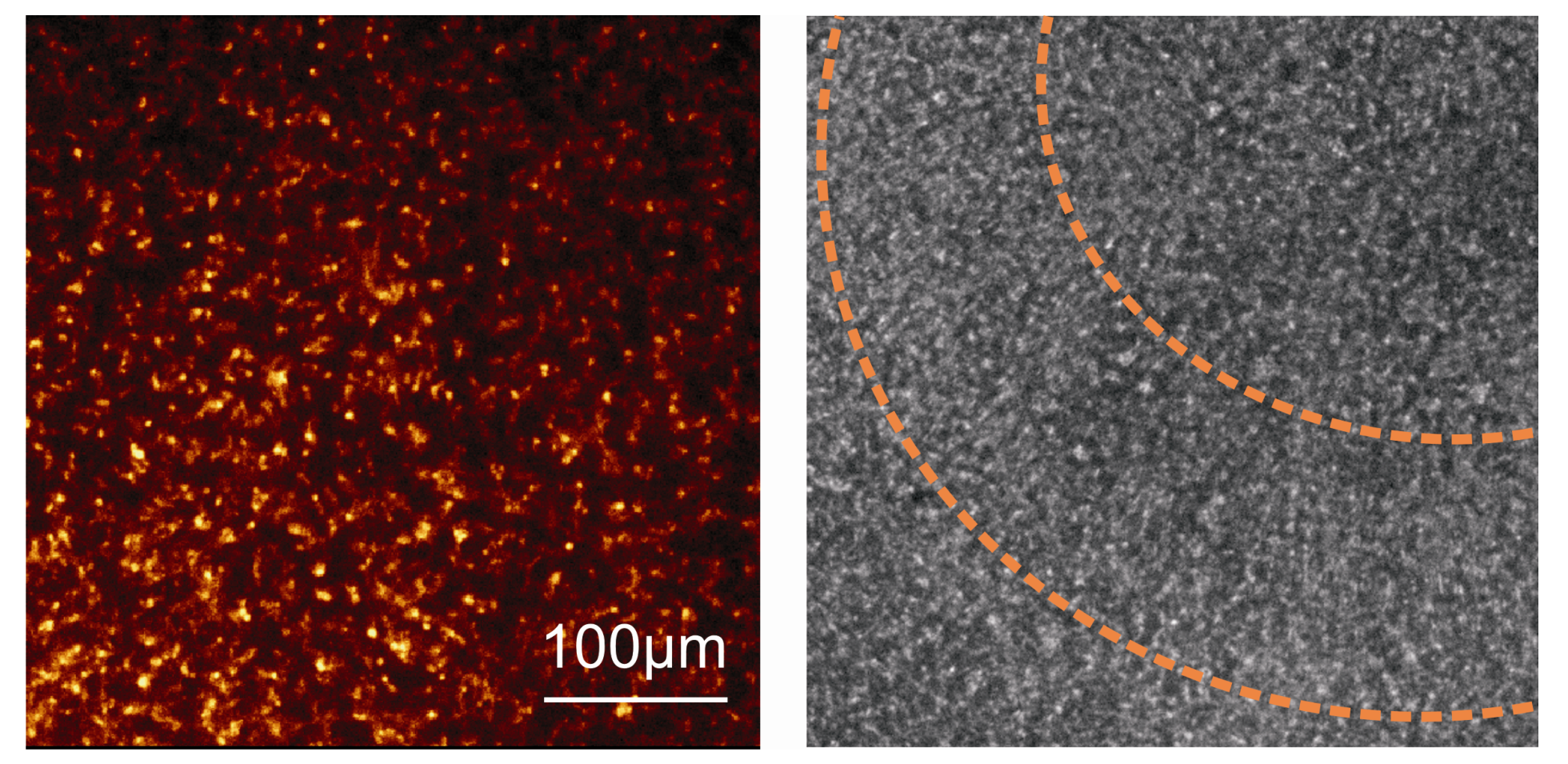

Supplement: Figure S2 and Video S2 — Effect of increased neuronal activity upon microglial movement: LPS. Here, Lipopolysaccharide (LPS) was added to the media 1 hour before time-lapse movie acquisition. LPS added to media increased neuronal excitability as shown in Figure 5 . Supplementary Figure S2 shows time zero frame of microglia to the left and matching phase image to the right with dotted lines marking the CA3 pyramidal neuron layer. (TIF, M2V) [file pone.0019294.s002.zip › Supplementary Figure 2.tif]

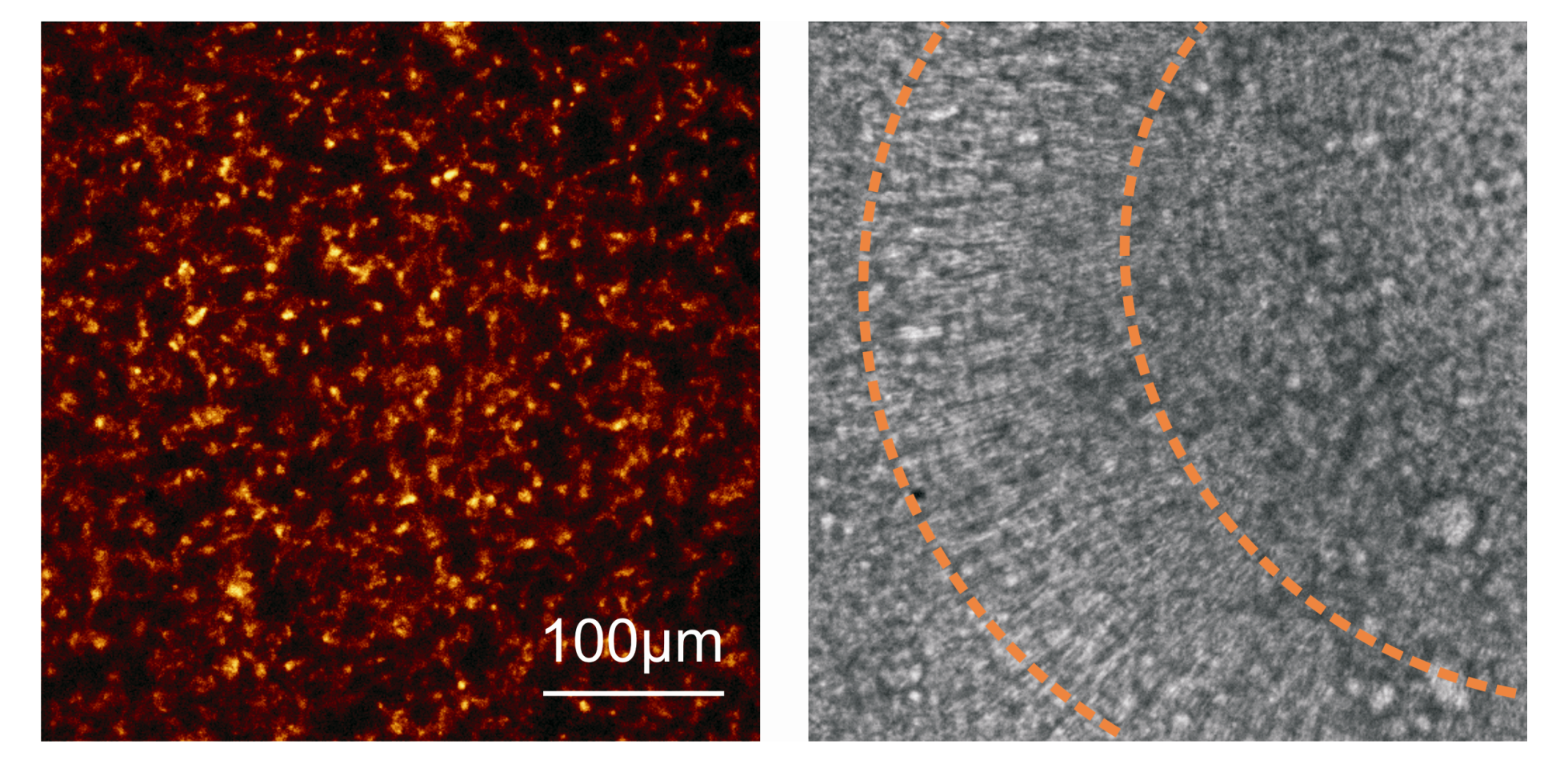

Supplement: Figure S3 and Video S3 — Effect of increased neuronal activity on microglial movement: cLTP. One hour before the start of the movie, the insert was placed in chemical long-term potentiation (cLTP) media. This cLTP protocol increased excitability as shown in Fig. 5 . Supplementary Figure S3 shows time zero frame of microglia to the left and matching phase image to the right with dotted lines marking the CA3 pyramidal neuron layer. (TIF, M2V) [file pone.0019294.s003.zip › Supplementary Figure 3.tif]

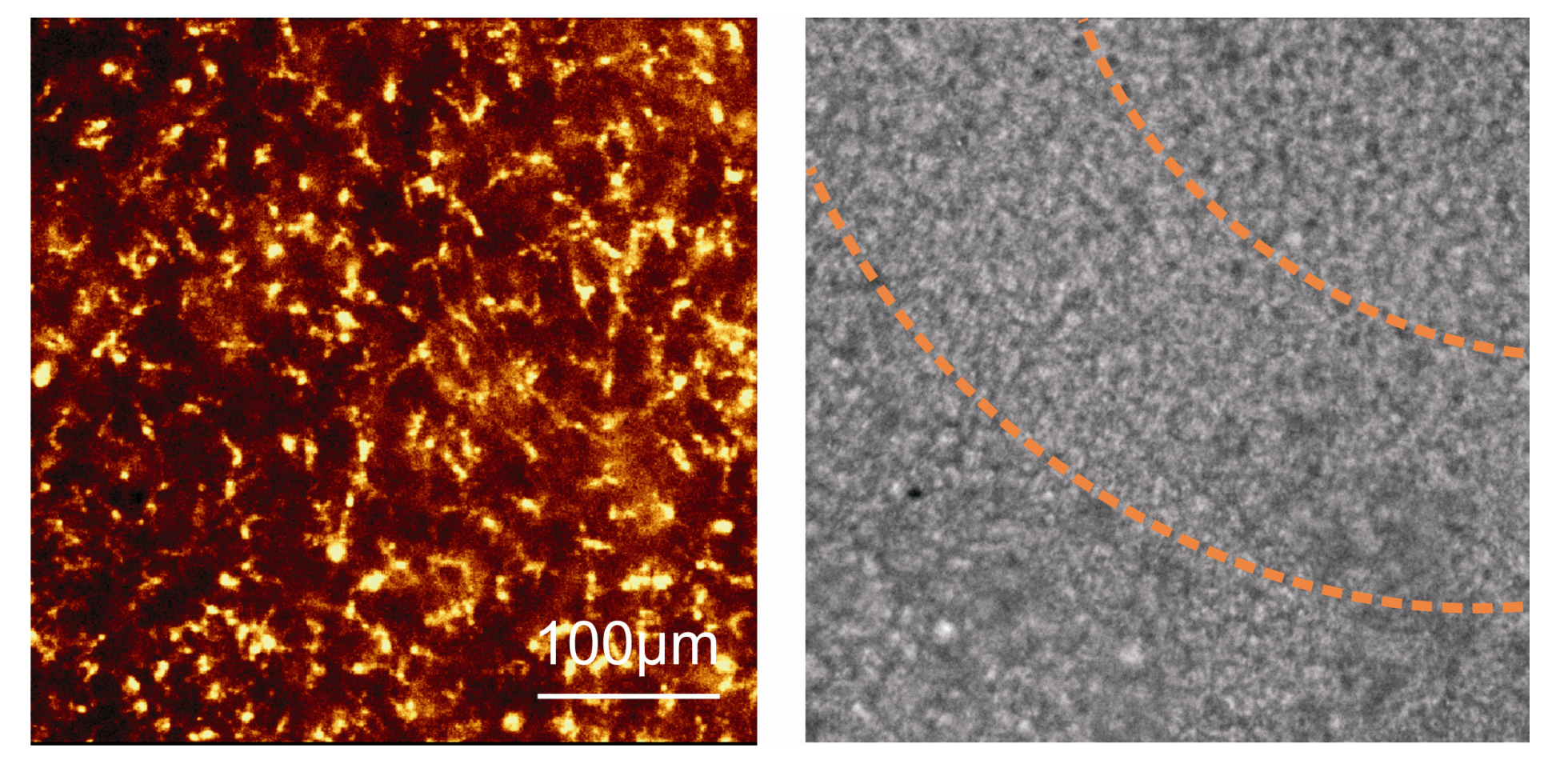

Supplement: Figure S4 and Video S4 — Effect of decreased neuronal activity upon microglial movement: TTX. Here, TTX was added to the media one hour before the start of the movie and was maintained throughout imaging. Supplementary Figure S4 shows time zero frame of microglia to the left and matching phase image to the right with dotted lines marking the CA3 pyramidal neuron layer. (TIF, M2V) [file pone.0019294.s004.zip › Supplementary Figure 4.tif]

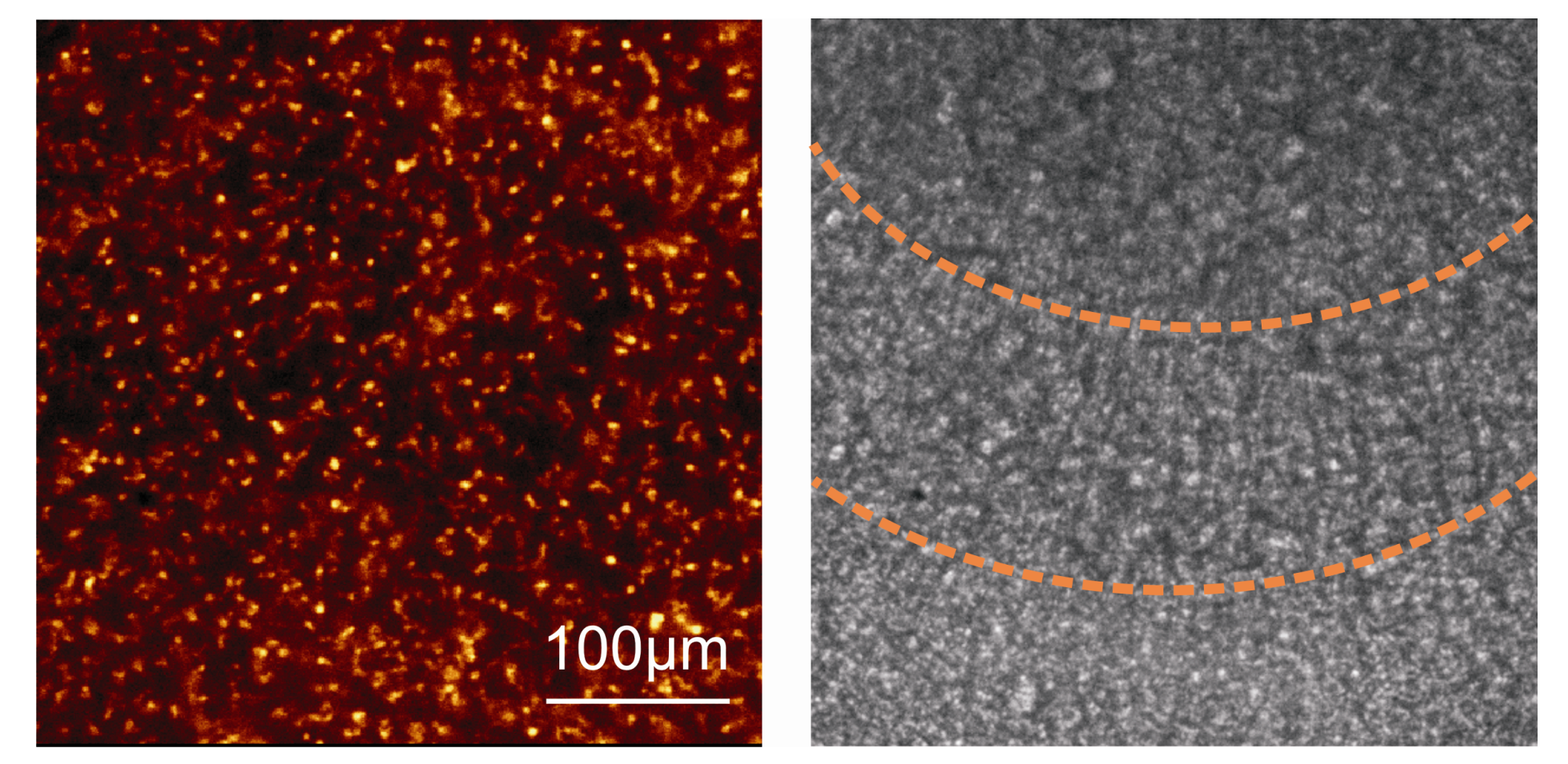

Supplement: Figure S5 and Video S5 — Effect of decreased neuronal activity upon microglial movement: Spreading depression. Spreading depression was induced 7 hours before the start of the movie. Supplementary Figure S5 shows time zero frame of microglia to the left and matching phase image to the right with dotted lines marking the CA3 pyramidal neuron layer. (TIF, M2V) [file pone.0019294.s005.zip › Supplementary Figure 5.tif]

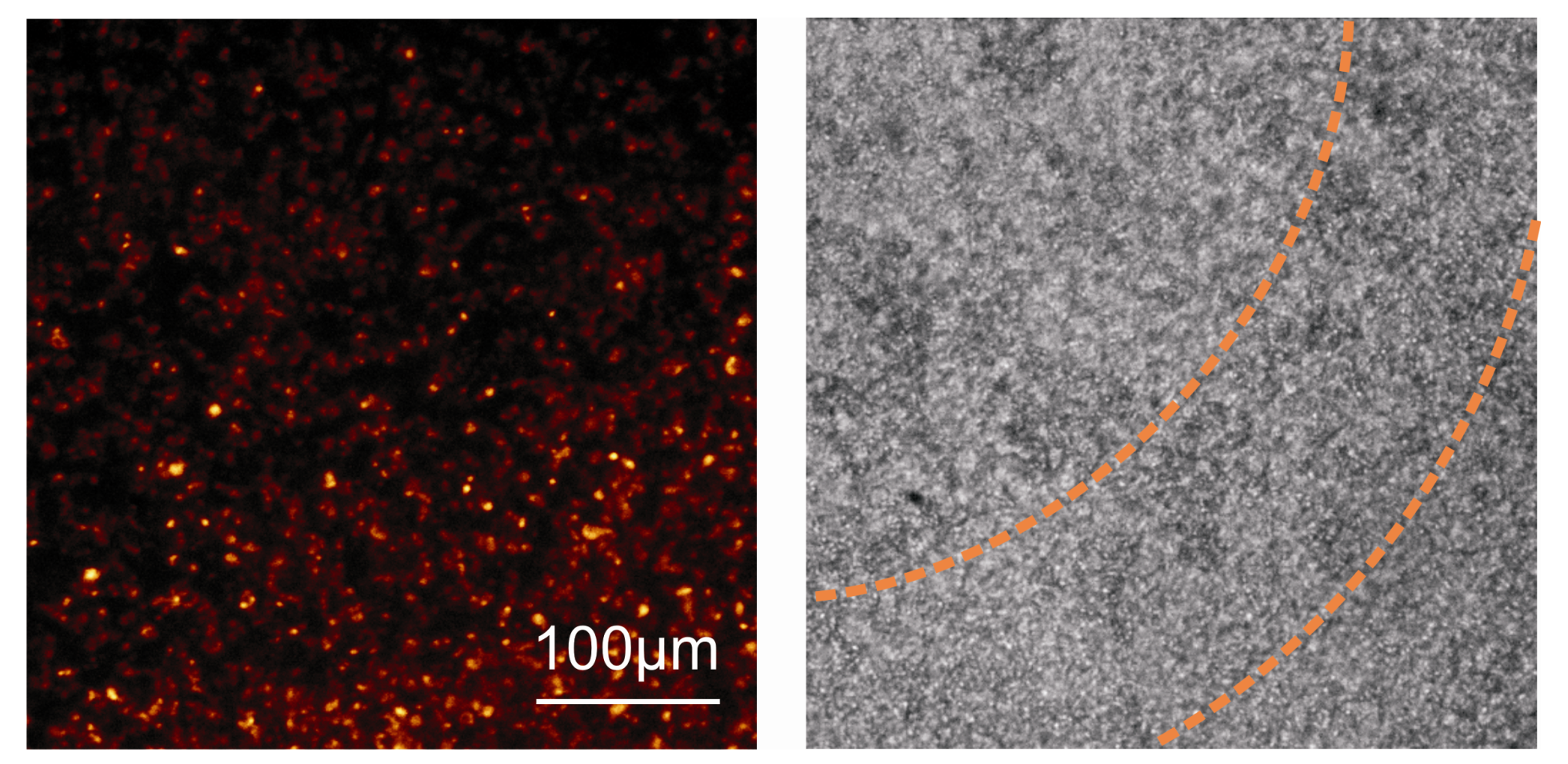

Supplement: Figure S6 and Video S6 — Addition of ATP and glutamate to TTX to mimic synaptic signaling. TTX was applied in the same manner as in the TTX-only movies, here with the addition of ATP and glutamate. Supplementary Figure S6 shows time zero frame of microglia to the left and matching phase image to the right with dotted lines marking the CA3 pyramidal neuron layer. (TIF, M2V) [file pone.0019294.s006.zip › Supplementary Figure 6.tif]
